# Definitive Fixation Timing and Orthopedic Events After Polytrauma With Long Bone Fractures: A Secondary Cohort Analysis

**DOI:** 10.7759/cureus.109122

**Published:** 2026-05-18

**Authors:** Eduard-Catalin Georgescu, Elisa-Georgiana Popescu

**Affiliations:** 1 Department 14 - Orthopedics and Intensive Care, Carol Davila University of Medicine and Pharmacy, Bucharest, ROU

**Keywords:** adverse orthopedic events, damage control orthopedics, definitive fixation, early appropriate care, long bone fractures, polytrauma, reoperation, secondary analysis, surgical site infection

## Abstract

Background: The timing of definitive fixation in polytrauma patients with long bone fractures must balance the benefits of early skeletal stabilization against the risks of operative stress in physiologically unstable patients. This study evaluated whether definitive fixation performed more than 24 hours after injury was associated with early orthopedic procedural events and broader adverse orthopedic outcomes.

Methods: We performed a secondary retrospective cohort analysis of 115 adult polytrauma patients with operatively treated long bone fractures. Early definitive fixation was defined as fixation within 24 hours (≤24 hours) after injury, and delayed fixation was defined as fixation more than 24 hours after injury. The primary endpoint was an early orthopedic procedural event, defined as deep surgical site infection within 90 days, reoperation by six months, or bone graft/biologic augmentation by six months. Secondary endpoints included a broader adverse orthopedic course, delayed union at 24 weeks, clinical union at 24 weeks, and nonunion at nine months. Continuous variables were compared using Welch's t-test. Categorical variables were compared using Pearson's chi-square test when expected cell counts were adequate and Fisher's exact test when expected cell counts were low. Logistic regression was used for exploratory adjusted analyses.

Results: Definitive fixation was performed within 24 hours in 33 patients (28.7%) and after 24 hours in 82 patients (71.3%). The primary procedural endpoint occurred in 20/115 patients (17.4%) and did not differ significantly between groups (4/33 patients (12.1%) vs. 16/82 patients (19.5%); chi-square = 0.895; p = 0.344). The broader adverse orthopedic course was more frequent after fixation beyond 24 hours (40/82 patients (48.8%) vs. 7/33 patients (21.2%); chi-square value = 7.400; p = 0.007). Delayed union at 24 weeks was also more frequent in the delayed fixation group (34/82 patients (41.5%) vs. 5/33 patients (15.2%); chi-square value = 7.269; p = 0.007). In adjusted analysis, fixation after 24 hours remained associated with the broader adverse orthopedic course (adjusted odds ratio = 3.00; 95% confidence interval = 1.09-8.25; z value = 2.124; p = 0.034), but not with the primary procedural endpoint.

Conclusions: In this secondary cohort analysis, definitive fixation beyond 24 hours was not independently associated with early orthopedic procedural events, but it was associated with a broader adverse orthopedic course driven mainly by delayed union. These findings support a cautious interpretation of fixation timing as both a potentially modifiable treatment factor and a marker of underlying physiologic and injury-pattern complexity.

## Introduction

Polytrauma is a complex clinical entity in which anatomic injury burden, hemorrhage, tissue hypoperfusion, systemic inflammation, and operative stress interact throughout the early clinical course [1]. Long bone fractures are clinically important within this context because they contribute to pain, blood loss, immobilization, inflammatory activation, and the need for staged or definitive orthopedic intervention. Injury Severity Score (ISS) and New Injury Severity Score (NISS) remain widely used tools for summarizing trauma burden, but decisions regarding fracture fixation require integration of both anatomic and physiologic information [2,3].

The timing of definitive fixation in polytrauma has evolved from early total care toward more individualized models. Damage control orthopedics prioritizes temporary stabilization in physiologically unstable patients to reduce the risk of additional operative stress, whereas Early Appropriate Care and Safe Definitive Surgery emphasize definitive stabilization once resuscitation and physiologic status permit safe surgery [4-10]. These approaches do not treat time alone as the only determinant of safety; rather, they interpret timing in relation to shock, acidosis, chest injury, traumatic brain injury, transfusion burden, and the risk of a second-hit response.

Delayed definitive fixation may, therefore, represent two related but distinct concepts. First, it may be a clinically necessary response to the complexity of the injury and physiologic instability. Second, it may prolong the interval before definitive mechanical stabilization, potentially influencing pain, mobilization, soft-tissue management, infection risk, reintervention, and fracture-healing trajectory. This creates a methodological challenge because delayed fixation may function both as a treatment-related exposure and as a marker of the clinical severity that prompted staged management [11-13].

Orthopedic adverse outcomes after polytrauma are heterogeneous. Early procedural events, including deep surgical site infection, reoperation, and augmentation with bone graft or biologics, are clinically relevant to the treating orthopedic surgeon. Healing-related outcomes, including delayed union and nonunion, are also important, but they are multifactorial and influenced by fixation stability, soft-tissue injury, systemic inflammation, infection, smoking, host biology, and local mechanical conditions [14-16]. Standardized radiographic follow-up systems such as the Radiological Union Scale for Tibia (RUST) and modified RUST may support more consistent assessment of fracture-healing progression [17-19].

The objective of the present study was to evaluate the association between definitive fixation timing and early orthopedic procedural events in polytrauma patients with long bone fractures. To reduce overlap with biomarker-focused or intensive-care-focused analyses of polytrauma outcomes, this secondary cohort analysis was restricted to operative timing and orthopedic events. We hypothesized that definitive fixation beyond 24 hours would be associated with a higher rate of a broader adverse orthopedic course, while early procedural event rates would be limited by the relatively low number of infections and reoperations.

## Materials and methods

Study design and setting

This retrospective observational study was designed as a secondary cohort analysis of an institutional polytrauma dataset comprising linked patient-level clinical variables, operative characteristics, early laboratory data, and radiographic follow-up assessments. The present analysis addressed a distinct clinical question focused on definitive fixation timing and orthopedic procedural events, rather than inflammatory biomarker trajectories or intensive care outcomes.

The study was conducted at the Clinical Emergency Hospital in Bucharest, Romania. The analytic cohort included 115 adult polytrauma patients with an index long bone fracture treated with definitive operative fixation. Patients were enrolled between January 2, 2022, and December 14, 2024. Follow-up data were available through May 26, 2025.

The retrospective research protocol for secondary analysis of routinely collected clinical, laboratory, operative, and follow-up data was approved by the Ethics Committee of the Clinical Emergency Hospital Bucharest (approval no. 3570, approval date: February 26, 2025). The requirement for individual informed consent was waived by the Ethics Committee because the study used routinely collected anonymized data. The study was reported in accordance with the Strengthening the Reporting of Observational Studies in Epidemiology recommendations [20].

Participants

Patients were eligible for inclusion if they had polytrauma, an index long bone fracture, available injury-severity data, and definitive operative fixation of the index fracture. Polytrauma was operationally represented by an ISS of at least 16 together with an operatively treated long bone fracture. All 115 patients in the analytic dataset had available time-to-definitive-fixation data and underwent definitive fixation.

Exposure variable

The primary exposure was time from injury to definitive fixation, recorded in hours. For the main analysis, patients were grouped as early definitive fixation, defined as definitive fixation performed within 24 hours (≤24 hours) after injury, and delayed definitive fixation, defined as definitive fixation performed more than 24 hours after injury. Time to definitive fixation was also analyzed as a continuous variable in logistic regression, using 12-hour increments for clinical interpretability.

Variables and clinical definitions

Baseline variables included age, sex, body mass index, smoking status, diabetes, obesity, osteoporosis, mechanism of injury, ISS, NISS, shock on admission, admission lactate, red blood cell units transfused within the first 24 hours, intensive care unit (ICU) admission, ICU length of stay, severe traumatic brain injury, severe chest injury, severe abdominal injury, associated pelvic ring injury, index fracture site, open-fracture status, multiple orthopedic injuries, temporary external fixation, definitive fixation type, and early weight-bearing protocol. For sex, entries coded as M and F were harmonized to male and female, respectively, before analysis.

ISS was calculated as the sum of the squares of the highest Abbreviated Injury Scale (AIS) severity score in each of the three most severely injured body regions. NISS was calculated as the sum of the squares of the three highest AIS scores regardless of body region [2,3]. Severe regional injuries were recorded when the AIS score for the relevant body region was at least 3.

Shock on admission was recorded as traumatic circulatory shock based on clinical documentation at presentation, including hypotension, tissue hypoperfusion, vasopressor requirement, or urgent blood-product resuscitation. The source dataset recorded shock as a binary admission variable and did not reliably separate hemorrhagic, neurogenic, obstructive, or septic shock subtypes. Admission lactate and transfusion burden were retained as baseline descriptors because they are clinically relevant markers of early physiologic derangement after trauma [21,22].

Radiographic assessment

Radiographic follow-up was performed using serial plain radiographs obtained during routine follow-up. Standard anteroposterior and lateral radiographs were used when appropriate for the fracture site. Computed tomography was not part of the predefined routine follow-up protocol and was not used as the primary modality for endpoint classification.

Fracture-healing assessment was based on the recorded radiographic healing category, healing score, number of bridged cortices, pain at the fracture site, and functional or weight-bearing status at follow-up. The modified RUST-based assessment considered cortical bridging/callus formation and fracture-line visibility across the evaluated cortices, consistent with established RUST and modified RUST concepts [17-19].

Outcomes

The primary endpoint was an early orthopedic procedural event, defined as at least one of the following: deep surgical site infection within 90 days, reoperation by six months, or bone graft/biologic augmentation by six months. This endpoint was selected to focus on early orthopedic events requiring procedural or surgical attention, rather than fracture-healing biology alone. Because the source dataset did not distinguish planned from unplanned bone grafts or biologic augmentations, this component was interpreted as a procedural escalation rather than a standalone complication.

Deep surgical site infection within 90 days was recorded when the clinical record documented deep infection involving the operative site or fixation region, including infection requiring operative management, deep antimicrobial treatment, or treating-surgeon documentation of deep infection. The 90-day window was selected to define an early postoperative deep infection outcome, in line with commonly used deep surgical site infection surveillance windows for procedures involving implants. However, fracture-related infection literature recognizes that later infections may occur beyond 90 days; therefore, this endpoint should not be interpreted as complete lifetime Fracture-Related Infection surveillance [23].

Secondary endpoints included each individual component of the primary endpoint, a broader adverse orthopedic course, delayed union at 24 weeks, clinical union at 24 weeks, and nonunion at nine months. The broader adverse orthopedic course was defined as the presence of at least one of the following: deep surgical site infection within 90 days, reoperation by six months, bone graft/biologic augmentation by six months, delayed union at 24 weeks, or nonunion at nine months. Delayed union and nonunion were therefore analyzed as secondary and exploratory healing-related outcomes, not as the primary endpoint of the study.

Statistical analysis

Continuous variables are presented as n and mean ± SD. Categorical variables are presented as analyzed n, event count, and percentage. For categorical variables with incomplete documentation in the routine clinical record, analyzed n represents the number of patients with available nonmissing data for that specific variable. Tables were structured so that each cell contains a single entry, and no table cells were intentionally left empty.

Continuous variables were compared using Welch's independent-samples t-test, and the corresponding t and p values were reported in separate columns. Categorical variables were compared using Pearson's chi-square test when all expected cell counts were at least 5. Fisher's exact test was used when any expected cell count was below 5. For Pearson's chi-square test, the chi-square value and p value were reported; for Fisher's exact test, the test statistic was recorded as not applicable, and the exact p value was reported. Categorical analyses used available-case denominators for each variable; missing observations were not imputed. No analysis of variance was performed because the main exposure was binary; therefore, F values were not applicable in the main tables.

Univariable logistic regression was performed for selected predictors of the primary procedural endpoint and the broader adverse orthopedic course. Given the limited number of primary procedural events, adjusted analysis for that endpoint was restricted to definitive fixation beyond 24 hours, ISS, and open-fracture status. For the broader adverse orthopedic course, multivariable logistic regression included definitive fixation beyond 24 hours, age, ISS, shock on admission, open fracture, and temporary external fixation. Effect sizes are reported as odds ratios with 95% confidence intervals, and logistic regression tables include z values and p values in separate columns. Statistical tests were two-sided, and p values below 0.05 were considered statistically significant.

## Results

The cohort included 115 patients. The mean age was 46.9 years, and 86 (74.8%) were male patients after harmonization of sex coding. The mean ISS was 25.4, and the mean NISS was 31.0. The mean time to definitive fixation was 31.2 hours, with a median of 30.5 hours.

Definitive fixation was performed within 24 hours in 33 patients (28.7%) and more than 24 hours after injury in 82 patients (71.3%). The primary early orthopedic procedural event occurred in 20 patients (17.4%). The broader adverse orthopedic course occurred in 47 patients (40.9%). Among individual outcomes, the most frequent was delayed union at 24 weeks (39 patients, 33.9%), followed by reoperation by six months (13 patients, 11.3%), nonunion at nine months (seven patients, 6.1%), bone graft/biologic augmentation by six months (six patients, 5.2%), and deep surgical site infection within 90 days (four patients, 3.5%).

Baseline and early clinical characteristics

Patients treated with definitive fixation more than 24 hours after injury had greater injury and physiologic complexity than those treated within 24 hours. The delayed fixation group had higher ISS and NISS, higher admission lactate, greater transfusion exposure, longer ICU length of stay, and a longer time to definitive fixation, as shown in Table 1.

**Table 1:** Continuous baseline and early clinical variables according to fixation timing Early fixation was defined as definitive fixation within 24 hours after injury. Delayed fixation was defined as definitive fixation more than 24 hours after injury. Continuous variables were compared using Welch's t-test BMI: body mass index; ICU: intensive care unit; ISS: Injury Severity Score; NISS: New Injury Severity Score; RBC: red blood cell

Variable	Early fixation, n	Early fixation, mean ± SD	Delayed fixation, n	Delayed fixation, mean ± SD	t value	p value
Age, years	33	47.7 ± 10.6	82	46.6 ± 13.3	0.445	0.658
BMI, kg/m^2^	33	27.3 ± 4.3	82	27.7 ± 4.1	-0.520	0.605
ISS	33	23.3 ± 3.7	82	26.3 ± 5.2	-3.510	<0.001
NISS	33	29.3 ± 4.4	82	31.7 ± 6.1	-2.418	0.018
Admission lactate, mmol/L	33	2.6 ± 1.0	78	3.2 ± 1.2	-2.690	0.009
RBC units for the first 24 hours	33	1.5 ± 1.4	82	2.4 ± 2.2	-2.718	0.008
ICU length of stay, days	33	3.4 ± 3.1	82	5.2 ± 3.6	-2.714	0.008
Time to definitive fixation, hours	33	16.4 ± 5.8	82	37.1 ± 10.2	-13.694	<0.001

Categorical baseline and operative variables also suggested greater clinical complexity in the delayed fixation group. Shock on admission, open fracture, and temporary external fixation were more frequent among patients treated after 24 hours, whereas sex, smoking status, diabetes, obesity, and severe associated regional injuries did not differ significantly between the groups (Table 2). Because several smoking-status and comorbidity variables had missing observations, Table 2 reports variable-specific analyzed denominators rather than assuming the full group sizes of 33 and 82 for every variable.

**Table 2:** Categorical baseline and operative variables according to fixation timing Categorical variables were compared using Pearson's chi-square test when all expected cell counts were at least 5 and Fisher's exact test when any expected cell count was below 5. For Fisher's exact test, the test statistic is listed as not applicable. The total early and delayed fixation groups included 33 and 82 patients, respectively. Denominators differ across selected variables because some smoking-status and comorbidity fields were incompletely documented in the routine clinical record; therefore, the analyzed n columns show the variable-specific number of patients with available nonmissing data. Percentages and statistical comparisons were calculated using these available-case denominators, and missing observations were not imputed AIS: Abbreviated Injury Scale; TBI: traumatic brain injury

Variable	Early analyzed, n	Early count	Early %	Delayed analyzed, n	Delayed count	Delayed %	Test used	Test statistic	p value
Male sex	33	23	69.7	82	63	76.8	Pearson chi-square	0.635	0.426
Current smoker	30	12	40.0	73	22	30.1	Pearson chi-square	0.935	0.333
Diabetes	32	2	6.2	78	5	6.4	Fisher exact	Not applicable	1.000
Obesity	33	8	24.2	82	29	35.4	Pearson chi-square	1.334	0.248
Osteoporosis	33	1	3.0	82	3	3.7	Fisher exact	Not applicable	1.000
Shock on admission	33	5	15.2	82	30	36.6	Pearson chi-square	5.106	0.024
Severe TBI (AIS ≥3)	33	5	15.2	82	17	20.7	Pearson chi-square	0.474	0.491
Severe chest injury (AIS ≥3)	33	10	30.3	82	33	40.2	Pearson chi-square	0.993	0.319
Severe abdominal injury (AIS ≥3)	33	6	18.2	82	17	20.7	Pearson chi-square	0.096	0.757
Associated pelvic ring injury	33	5	15.2	82	9	11.0	Fisher exact	Not applicable	0.539
Open fracture	33	2	6.1	82	23	28.0	Pearson chi-square	6.687	0.010
Multiple orthopedic injuries	33	7	21.2	82	32	39.0	Pearson chi-square	3.331	0.068
Temporary external fixation	33	1	3.0	82	24	29.3	Pearson chi-square	9.521	0.002

Orthopedic outcomes

Orthopedic outcomes by fixation timing are summarized in Table 3, with the predefined assessment window for each endpoint. The primary early orthopedic procedural endpoint did not differ significantly between the groups, whereas the broader adverse orthopedic course and delayed union at 24 weeks were more frequent among patients treated more than 24 hours after injury.

**Table 3:** Orthopedic outcomes according to fixation timing The early orthopedic procedural event was defined as deep SSI within 90 days, reoperation by six months, or bone graft/biologic use by six months. Because planned and unplanned augmentation could not be distinguished in the source dataset, bone graft/biologic use was interpreted as procedural escalation. The adverse orthopedic course was defined as the early procedural endpoint, delayed union at 24 weeks, or nonunion at nine months. Categorical variables were compared using Pearson's chi-square test when all expected cell counts were at least 5 and Fisher's exact test when any expected cell count was below 5. For Fisher's exact test, the test statistic is listed as not applicable. Percentages and statistical comparisons were calculated using the analyzed n values shown in the table SSI: surgical site infection

Variable	Assessment window	Early analyzed, n	Early count	Early %	Delayed analyzed, n	Delayed count	Delayed %	Test used	Test statistic	p value
Early orthopedic procedural event	Up to 6 months	33	4	12.1	82	16	19.5	Pearson chi-square	0.895	0.344
Deep SSI	90 days	33	0	0.0	82	4	4.9	Fisher exact	Not applicable	0.324
Reoperation	6 months	33	2	6.1	82	11	13.4	Fisher exact	Not applicable	0.343
Bone graft/biologic use	6 months	33	2	6.1	82	4	4.9	Fisher exact	Not applicable	1.000
Adverse orthopedic course	Up to 9 months	33	7	21.2	82	40	48.8	Pearson chi-square	7.400	0.007
Delayed union	24 weeks	33	5	15.2	82	34	41.5	Pearson chi-square	7.269	0.007
Clinical union	24 weeks	33	28	84.8	82	48	58.5	Pearson chi-square	7.269	0.007
Nonunion	9 months	33	2	6.1	82	5	6.1	Fisher exact	Not applicable	1.000

Regression analysis

In univariable logistic regression, definitive fixation beyond 24 hours was not significantly associated with the primary early orthopedic procedural event. In a limited adjusted model including fixation beyond 24 hours, ISS, and open-fracture status, fixation beyond 24 hours remained nonsignificant for the primary procedural endpoint.

For the broader adverse orthopedic course, definitive fixation beyond 24 hours was associated with higher odds in univariable analysis. In the multivariable model adjusted for age, ISS, shock on admission, open fracture, and temporary external fixation, fixation beyond 24 hours and shock on admission remained independently associated with the broader adverse orthopedic course.

Logistic regression analyses are presented in Table 4. Definitive fixation beyond 24 hours was not independently associated with the primary early procedural orthopedic endpoint, but it remained associated with the broader adverse orthopedic course after adjustment for clinically relevant covariates.

**Table 4:** Logistic regression for early orthopedic procedural events and broader adverse orthopedic course The multivariable model for early orthopedic procedural events included definitive fixation beyond 24 hours, ISS per five points, and open-fracture status. The multivariable model for adverse orthopedic course included definitive fixation beyond 24 hours, age per 10 years, ISS per five points, shock on admission, open-fracture status, and temporary external fixation CI: confidence interval; ISS: Injury Severity Score

Outcome	Model	Predictor	n	Events	Odds ratio	CI lower	CI upper	z value	p value
Early orthopedic procedural event	Univariable	Definitive fixation >24 hours	115	20	1.76	0.54	5.72	0.937	0.349
	Multivariable	Definitive fixation >24 hours	115	20	1.38	0.39	4.88	0.497	0.619
	Multivariable	ISS per 5 points	115	20	1.24	0.76	2.05	0.858	0.391
	Multivariable	Open fracture	115	20	1.53	0.50	4.70	0.749	0.454
Adverse orthopedic course	Univariable	Definitive fixation >24 hours	115	47	3.54	1.38	9.06	2.634	0.008
	Univariable	Time to fixation per 12 hours	115	47	1.38	0.97	1.97	1.803	0.071
	Multivariable	Definitive fixation >24 hours	115	47	3.00	1.09	8.25	2.124	0.034
	Multivariable	Age per 10 years	115	47	1.04	0.76	1.43	0.251	0.802
	Multivariable	ISS per 5 points	115	47	0.77	0.48	1.25	-1.043	0.297
	Multivariable	Shock on admission	115	47	3.02	1.09	8.38	2.124	0.034
	Multivariable	Open fracture	115	47	0.89	0.30	2.63	-0.207	0.836
	Multivariable	Temporary external fixation	115	47	1.75	0.55	5.54	0.944	0.345

## Discussion

The main finding of this secondary cohort analysis is that definitive fixation beyond 24 hours was not significantly associated with the primary endpoint of early orthopedic procedural events, defined as deep surgical site infection, reoperation, or bone graft/biologic augmentation. However, fixation beyond 24 hours was associated with a broader adverse orthopedic course, mainly driven by delayed union at 24 weeks. This distinction is important because it separates early procedural events from later healing-related outcomes and avoids interpreting all adverse orthopedic outcomes as biologically equivalent.

The absence of a statistically significant association between fixation beyond 24 hours and early procedural events should be interpreted cautiously. Deep surgical site infection, reoperation, and bone graft/biologic augmentation were relatively infrequent in this cohort, limiting statistical power for the primary endpoint. The numerically higher procedural event rate in the delayed fixation group may still be clinically relevant, but this study cannot establish a definitive independent association for procedural complications alone.

The association between delayed fixation and the broader adverse orthopedic course is clinically plausible but should not be interpreted as proof of causality. Patients treated after 24 hours had higher injury severity, higher lactate, greater transfusion exposure, longer ICU stay, more frequent shock, more frequent open fractures, and greater use of temporary external fixation. These differences suggest that delayed fixation was at least partly a marker of physiologic instability and injury-pattern complexity rather than a simple discretionary delay.

This interpretation aligns with contemporary concepts in polytrauma care. Earlier Early Appropriate Care studies supported definitive stabilization within 24 hours in physiologically suitable patients, whereas damage control orthopedics and Safe Definitive Surgery frameworks emphasize that fixation timing must be adapted to shock, resuscitation status, concomitant injuries, and operative risk [4-10]. Our findings are consistent with this literature because they suggest that delayed fixation identifies a clinically more complex subgroup rather than proving that 24 hours is an absolute biological threshold.

The secondary finding related to delayed union should also be interpreted in an integrated clinical framework. Fracture healing after polytrauma depends on local mechanical stability, soft-tissue injury, perfusion, infection, systemic inflammation, host factors, and rehabilitation conditions [14-16,24]. A longer interval before definitive fixation may prolong the period before optimal mechanical stabilization, but it may also identify patients with worse local soft-tissue conditions, open fractures, shock, transfusion exposure, and staged management needs. Thus, time to definitive fixation may function as a practical risk marker for closer follow-up rather than as an isolated causal determinant of delayed union.

From a practical standpoint, the results suggest that patients requiring fixation beyond 24 hours may warrant more structured orthopedic surveillance even when early procedural complications do not occur. This may include careful wound monitoring, early recognition of fixation-related problems, standardized radiographic follow-up, and attention to delayed progression of union. The association with shock on admission in the adjusted model further supports the need to interpret orthopedic risk in relation to early physiologic derangement, rather than through operative timing alone.

This study has several limitations. First, the retrospective design prevents causal inference. Second, treatment after 24 hours was not randomly assigned and was likely influenced by physiologic instability, open-fracture status, soft-tissue injury, injury complexity, and surgeon judgment. Third, the 24-hour threshold was selected as a pragmatic and literature-informed cutoff for early definitive fixation; it should not be interpreted as a definitive biological threshold for delayed union or nonunion. Fourth, the number of early procedural events was limited, reducing power for infection, reoperation, and bone-graft outcomes. Fifth, the dataset recorded bone graft/biologic augmentation by six months but did not distinguish planned from unplanned use; therefore, this component should be interpreted as procedural escalation rather than as a standalone complication. Sixth, deep surgical site infection was assessed within a 90-day early postoperative window and should not be interpreted as complete fracture-related infection surveillance; later infections, including possible hematogenous infections or infections presenting as nonunion, could not be fully evaluated. Seventh, shock was recorded as a binary admission variable, and the dataset did not reliably separate hemorrhagic, neurogenic, obstructive, or septic shock subtypes. Eighth, the broader adverse orthopedic course combines procedural events and healing-related outcomes; this improves event capture but combines outcomes with different mechanisms. Ninth, the dataset did not include all potentially relevant details, such as reduction quality, fixation construct characteristics, operative duration, vasopressor exposure, mechanical ventilation duration, or granular soft-tissue severity. Finally, this was a single-center secondary analysis, which may limit generalizability.

Despite these limitations, the study has strengths. It evaluates a clinically relevant cohort of polytrauma patients with long bone fractures, uses a pragmatic 24-hour threshold for definitive fixation, separates early procedural events from broader healing-related outcomes, transparently identifies the analysis as secondary, and reports statistical test values in accordance with editorial requirements. Future prospective multicenter cohorts with standardized definitions of shock, fixation timing, radiographic follow-up, and fracture-related infection would be useful for further evaluating these associations and determining whether timing-based risk stratification improves outcomes.

## Conclusions

In this secondary retrospective cohort analysis of polytrauma patients with long bone fractures, definitive fixation beyond 24 hours was not significantly associated with early orthopedic procedural events, including deep surgical site infection, reoperation, or bone graft/biologic augmentation. However, delayed fixation was associated with a broader adverse orthopedic course, mainly driven by delayed union at 24 weeks. These findings support individualized fixation timing based on physiologic readiness and identify patients treated after 24 hours as a subgroup that may benefit from closer orthopedic follow-up.
